# Case of Puerperal Convulsions

**Published:** 1860

**Authors:** Wm. H. Byford

**Affiliations:** Prof. of Obstetrics, etc., in Medical Department of Lind University


*Case of Puerperal Convulsions.* Reported by Wm. H. Byford, M. D., Prof. of Obstetrics, etc., in Medical Department of Lind University.

Mrs. R., a delicate but healthy woman, of nervous sanguine temperament, aged 27 years, was confined August 5th, 1859, about 9 o'clock, of a female child, which she thought was two weeks too soon. Although she enjoyed what she considered excellent health during this, her second pregnancy, for the last two or three weeks her feet and legs were edematous; she had some edema of the face and perhaps elsewhere, and was annoyed by frequent small discharges of urine, more, I should judge, than usual. Her labor, according to the report of her very intelligent physician, Dr. Parker, was in no wise extraordinary, and up to 7 o'clock, A. M., on the sixth, she seemed to be doing remarkably well. About this time she suddenly complained of a distressing pain in one temple and eye, and total blindness. These symptoms in a few moments were followed by a paroxysm of convulsions. Dr. Parker soon arrived, and prescribed a teaspoonful of Hoffman's Anodyne, and twenty drops of tincture of hyosciamus every two hours. The convulsions recurred at 12 o'clock, M., with increased force. In consultation with Drs. Davis and Parker, prescribed teaspoonful of Fl. ext. scutellaria, twenty drops tincture hyosciamus, and five grains of iodide of potassium every two hours. At 5 o'clock, P. M., convulsions again returned, when, as further council was desired, I was called.

In this consultation it was agreed that the patient should have the surface thoroughly and frequently sponged with tepid vinegar, and at the approach of a paroxysm to have chloroform by inhalation, until the symptoms should be subdued, and continue the mixture. It was also agreed that Dr. P. should remain with her until 10 o'clock, when I was to relieve him for the night.—At quarter past 10, and just after Dr. P. had left me alone with the patient, a fearful paroxysm returned, and again at half-past ten o'clock. I tried the chloroform, but its inhalation was resisted with such wild violence that I thought it best to desist, so that each of these two paroxysms proceeded uninterruptedly, and I was assured that although the effort had been made to administer it under other circumstances, it disagreed with her so much that its effects could never be completely induced. I now added one-eighth of a grain of sul. morphia to each portion of the mixture. The administration of the medicine now, as it had been before, was very imperfect on account of the energetic exertion with which she resisted it, so that it is doubtful whether the full effect was at any time produced. At quarter-past 3 o'clock, A. M., the convulsions returned, and the paroxysm was repeated in fifteen minutes afterwards.

It seemed to me that as yet the symptoms had not been in the least degree influenced by the remedies, and I determined to try the effect of morphia in pretty full doses. I accordingly gave her a quarter of a grain, dissolved in a small quantity of water in a spoon, every two hours, and by holding her nose and literally drenching her, had the satisfaction to see the whole of it swallowed. This treatment, with the vinegar sponging, was continued until 6, P. M., when there being no return of the convulsions, it was deemed advisable to completely withdraw the anodyne, and give, in its place, a teaspoonful of sweet spirits of nitre every two hours. At 9 o'clock, A. M., of the 8th, consciousness had partially returned, and the bowels had moved spontaneously three times. Everything, so far as we could judge, was favorable to speedy recovery. Between 12 and 1 o'clock, P. M., a paroxysm of irritative fever occurred, in which the pulse rose to about 120 in a minute, and there was considerable heat of the skin. The fever subsided about 3 o'clock, A. M., on the 9th, with profuse perspiration.

During the paroxysm she took a pill containing two grains of calomel and one grain of opium every two hours. Fever returned at 3 o'clock in the evening. At 12 o'clock, Noct. Med, she began taking a pill of two and a half grains of sul. quinine every two hours. She took during the night and forepart of the day of the 10th, eighteen grains of quinine, which had the effect to prevent the recurrence of the fever. The patient pretty rapidly recovered from this time forward.

I regard this case as one clearly of uræmic eclampsia, and do not believe that there was anything of the apoplectic congestion, which used to be considered the invariable condition of the nervous centres in purperal convulsions. One thing I could not fail to see, viz: the increased restlessness before the paroxysms, and at the same time heard tumultuous borborygma intestinalis. From these circumstances, with direction of the hands to the abdomen, I believed the restlessness arose from pain in that region, which in the highly excitable state of the nervous centres, produced by the toxemia, gave rise to the paroxysm. To express it otherwise, the intestinal irritation acted as an excentric excito-motor upon the nervous centres, through the reflex system of Sir Marshall Hall, while the irritability of the whole nervous mass being greatly exaggerated by the uræmia, convulsions were thus easily induced.

We sought in the sul. of morphia, through its anodyne influence, an extinguisher of the exciting cause and a paliative for the nervous irritability of the nervous system, arising from the toxemia; and I think, as will be apparent in the above narrative of the case, not in vain. For several hours after beginning the treatment, we anxiously watched the respiration and pulse for any circumstance that might arise indicating narcotism or congestion of the brain. No symptoms, however, were perceptible that gave us any uneasiness in these respects. The respiration did not become suspiratory nor intermittent, nor the pulse slower and fuller. On the contrary, the respiratory function was performed regularly and calmly, and the pulse being irritable and wiry, became slower, softer, and more healthy.

Viewing these fearful attacks of puerperal females by the improved and increased light of science, emanating from the number of enlightened modern pathologists and chemico-pathologists now in the field, I cannot but think we will find in our anodynes, as well as our anæsthetics, more important auxiliaries in their treatment than has hitherto been supposed.

Before closing my remarks on the above case, I cannot forbear to say, that while there are many cases that will bear and require depletion even to a considerable extent, opiates are not to be dreaded as much even in these cases as we have been taught by many able authors of past and present times.

I beg the profession to observe that here is one case in which the anodyne was the only efficient treatment used to overcome the convulsions, and it resulted successfully. It may be said, and perhaps not without justice, that the patient simply recovered without being cured; but I am persuaded, from the effects of the morphia in this case, to remember it in future as one of the remedies in puerperal convulsions.

There is one feature in eclampsia that seems to me to prove the reflex element is often quite active, and that is, the periodical regularity of the return of the paroxysms. The measured intervals in very many cases enable us to predict within a few moments the time of their recurrence. It is in these cases we may most certainly benefit by the interrupting influence of the anodynes and anæsthetics. We should anticipate them by preoccupying the nervous system with an opposing condition.—*Chicago Medical Examiner.*

